# Cardiometabolic Risk Profiles Associated with Chronic Gastrointestinal Symptoms in Adults: A Cross-Sectional Exploratory Analysis Using Routine Clinical Markers

**DOI:** 10.3390/nu18060892

**Published:** 2026-03-12

**Authors:** Ramona Alina Tomuța, Roxana Daniela Brata, Marc Cristian Ghitea, Evelin Claudia Ghitea, Maria Flavia Gîtea, Timea Claudia Ghitea, Florin Banica

**Affiliations:** 1 Doctoral School of Biological and Biomedical Sciences, University of Oradea, 1 University Street, 410087 Oradea, Romania; yasmine_tomas@yahoo.com; 2 Department of Medical Disciplines, Faculty of Medicine and Pharmacy, University of Oradea, 1 University Street, 410087 Oradea, Romania; 3 Faculty of Medicine and Pharmacy, University of Oradea, 410068 Oradea, Romania; ghitea.marccristian@student.uoradea.ro (M.C.G.); ghitea.evelinclaudia@student.uoradea.ro (E.C.G.); gitea.mariaflavia@student.uoradea.ro (M.F.G.); 4 Pharmacy Department, Faculty of Medicine and Pharmacy, University of Oradea, 1 University Street, 410087 Oradea, Romania; florinbanica1@gmail.com

**Keywords:** gastrointestinal symptoms, cardiometabolic risk, dyslipidemia, fasting glucose, triglyceride/HDL ratio, composite metabolic stress score, routine clinical biomarkers, cross-sectional study

## Abstract

Background: Persistent gastrointestinal (GI) symptoms are common in adults and are often considered functional conditions. Emerging evidence suggests that gastrointestinal function may be intertwined with systemic metabolic regulation, yet the association between chronic GI symptoms and cardiometabolic risk assessed using routine clinical biomarkers remains insufficiently explored. Methods: In this cross-sectional observational study, 93 adults were consecutively enrolled during routine clinical evaluations. Anthropometric parameters, fasting plasma glucose, glycated hemoglobin (HbA1c), lipid profile, and blood pressure were assessed. Participants were classified as having persistent gastrointestinal symptoms (GI+) or being asymptomatic (GI−) based on symptom duration and clinical documentation. A composite metabolic stress score, derived from routinely available biomarkers, was used to summarize multidimensional cardiometabolic burden. Group comparisons and correlation analyses were performed using non-parametric methods. Results: Participants with persistent gastrointestinal symptoms exhibited higher triglyceride levels, lower HDL-cholesterol concentrations, and higher fasting plasma glucose compared with asymptomatic individuals (all *p* < 0.05). The composite metabolic stress score was significantly higher in the GI+ group, indicating greater overall cardiometabolic burden, while body mass index and HbA1c did not differ significantly between groups. Conclusions: Persistent gastrointestinal symptoms were associated with an unfavorable cardiometabolic profile characterized by atherogenic dyslipidemia and impaired fasting glycemia. An exploratory composite metabolic stress score based on routine clinical biomarkers effectively summarized this pattern. These findings support the biological plausibility of shared metabolic vulnerability between gastrointestinal symptom burden and cardiometabolic risk and highlight the need for longitudinal and mechanistic studies incorporating objective gastrointestinal and metabolic biomarkers.

## 1. Introduction

Cardiometabolic disorders represent a major global health burden and are closely linked to obesity, dyslipidemia, impaired glucose metabolism, and elevated blood pressure [1,2,3]. These conditions often coexist and interact across multiple physiological systems, contributing to increased cardiovascular morbidity and mortality [4,5,6]. While the metabolic consequences of obesity and insulin resistance have been extensively studied, less attention has been paid to the potential clinical relevance of persistent gastrointestinal symptoms within cardiometabolic risk assessment [7,8,9].

Gastrointestinal symptoms such as bloating, abdominal discomfort, altered bowel habits, and postprandial intolerance are highly prevalent in adult populations and are frequently classified as functional disorders [10,11,12,13]. However, emerging evidence suggests that gastrointestinal function may be closely intertwined with systemic metabolic regulation. Experimental and clinical studies have highlighted the role of the gut environment—including dietary factors, microbial metabolism, and intestinal barrier integrity—in shaping lipid metabolism, glucose homeostasis, and low-grade systemic inflammation [14,15,16,17]. Within this context, gastrointestinal symptoms may represent a clinical manifestation of broader metabolic vulnerability rather than isolated functional complaints [18,19].

Most research exploring gut–metabolism interactions relies on advanced microbiome, imaging, or molecular biomarkers. While scientifically informative, these approaches are rarely available in routine clinical nutrition or primary care. Therefore, there is limited evidence on whether simple, routinely measured clinical markers can capture metabolic patterns associated with gastrointestinal symptom burden. This study addresses that practical gap by testing whether routinely available biomarkers can reflect multidimensional metabolic vulnerability in individuals reporting persistent GI symptoms [20].

Previous research has demonstrated associations between metabolic disorders and gastrointestinal conditions, including non-alcoholic fatty liver disease, metabolic syndrome, and type 2 diabetes, in which digestive symptoms are commonly reported. Dyslipidemia and impaired glycemic control, in particular, have been linked to alterations in gastrointestinal motility, bile acid signaling, and gut-derived inflammatory pathways. Nevertheless, most studies investigating gut–metabolism interactions rely on specialized biomarkers, imaging, or microbiome analyses that are not routinely available in clinical practice. Although objective gastrointestinal biomarkers are essential for diagnosis, they are not always available in routine nutritional or primary care settings. Therefore, symptom-based information may serve as a complementary clinical signal rather than a diagnostic substitute [21,22,23,24].

In everyday clinical settings, cardiometabolic risk is primarily evaluated using accessible biomarkers such as body mass index, fasting plasma glucose, lipid profile, and blood pressure measurements. Whether gastrointestinal symptom burden is associated with adverse patterns in these routine metabolic markers remains insufficiently explored. Clarifying this relationship could provide clinically relevant insights into early metabolic risk stratification and highlight the importance of integrated symptom-based assessment [25,26,27,28].

Therefore, the aim of this study was to examine the association between persistent gastrointestinal symptoms and cardiometabolic risk profiles in adults using routinely available clinical biomarkers. Specifically, we compared anthropometric, glycemic, lipid, and cardiovascular parameters between participants with and without chronic gastrointestinal symptoms and summarized multidimensional metabolic burden using an exploratory composite metabolic stress score. By adopting a symptom-based and biomarker-driven approach, this study seeks to contribute to a more integrated understanding of gastrointestinal symptoms within the broader framework of cardiometabolic health.

## 2. Materials and Methods

### 2.1. Study Design and Population

This observational, cross-sectional study included 93 adult participants consecutively enrolled between 2023 and 2024 during routine clinical nutrition consultations. The study population represents a convenience sample reflecting real-world cardiometabolic and nutritional evaluation.

Inclusion criteria were: age ≥18 years and availability of complete anthropometric, biochemical, and clinical data. Exclusion criteria were: acute liver disease, known chronic liver disease of non-metabolic origin, and chronic alcohol consumption (>20 g/day for women and >30 g/day for men).

The study intentionally reflects a real-world clinical nutrition population, where overweight and obesity are highly prevalent. The aim was not population representativeness but exploratory assessment within a metabolically at-risk group.

All participants were evaluated in a standardized clinical setting, and data were collected from routine medical records and clinical assessments. The study is reported in accordance with STROBE recommendations for observational studies.

Sociodemographic characteristics (age and sex distribution) were recorded for all participants, whereas detailed lifestyle variables such as diet, smoking status, and physical activity were not systematically collected in the clinical records.

### 2.2. Definition of Gastrointestinal Symptoms

Persistent gastrointestinal symptoms (GI+) were defined based on self-reported digestive complaints lasting at least three months. Symptoms considered in the classification included bloating, abdominal discomfort, altered bowel habits (constipation or diarrhea), and postprandial distress, as documented during routine clinical consultations.

Gastrointestinal symptoms were assessed using a semi-structured clinical interview conducted during routine clinical nutrition consultations, focusing on common digestive complaints. Participants were classified as GI+ when at least one persistent symptom was reported for ≥3 months and recorded in the clinical documentation.

No standardized diagnostic questionnaire (e.g., Rome criteria) was applied because the study aimed to capture patient-reported symptom burden in a real-world clinical consultation context rather than formally diagnosed gastrointestinal disorders.

### 2.3. Clinical and Biochemical Measurements

The following variables were recorded for each participant:

- Anthropometric data: body mass index (BMI, kg/m^2^), calculated as weight divided by height squared.
- Glycemic parameters: fasting plasma glucose (mg/dL) and glycated hemoglobin (HbA1c, %).
- Lipid parameters: triglycerides (TG, mg/dL), high-density lipoprotein cholesterol (HDL-C, mg/dL), and low-density lipoprotein cholesterol (LDL-C, mg/dL).
- Cardiovascular parameters: systolic (SBP) and diastolic blood pressure (DBP), measured in mmHg using standard clinical procedures. Blood pressure was analyzed separately to avoid overweighting cardiovascular parameters and to maintain a score focused on metabolic domains.

All biochemical analyses were performed using standardized laboratory methods as part of routine clinical care.

### 2.4. Development of the Composite Metabolic Stress Score

To summarize cardiometabolic burden using accessible biomarkers, an exploratory Composite Metabolic Stress Score was constructed. Metabolic stress refers here to the clustering of dyslipidemia, adiposity, and glycemic alterations. The score integrates four routinely measured variables known to reflect cardiometabolic and hepatic-metabolic load:

- Body mass index (BMI);
- Triglycerides (TG);
- Inverse HDL-cholesterol (−HDL-C);
- Glycated hemoglobin (HbA1c).

Each variable was standardized using z-score transformation (mean = 0, standard deviation = 1). The composite score was calculated as the arithmetic mean of these standardized values, with higher scores indicating greater cardiometabolic stress.

Composite Score = (zBMI + zTG + z(−HDL-C) + zHbA1c)/4

This composite score is exploratory in nature and was designed to summarize multidimensional metabolic burden rather than to serve as a validated diagnostic index. Selected components reflect core domains of cardiometabolic risk (adiposity, lipid metabolism, glycemic control), consistent with commonly used metabolic syndrome frameworks [29].

### 2.5. Data Processing

All variables were screened for plausibility and missing values. Continuous variables with less than 5% missing data were imputed using the median. Variables included in the composite score were standardized prior to score construction.

### 2.6. Statistical Analysis

Statistical analyses were performed using SPSS version 30 (IBM Corp., Armonk, NY, USA). Continuous variables were summarized as mean ± standard deviation (SD) or median with interquartile range (IQR), depending on distribution. Comparisons between participants with (GI+) and without (GI−) gastrointestinal symptoms were performed using the Mann–Whitney U test for continuous variables and the chi-square test for categorical variables. Associations between metabolic parameters and the composite metabolic stress score were evaluated using Spearman’s rank correlation coefficient (ρ). A two-sided *p*-value < 0.05 was considered statistically significant.

### 2.7. Ethical Approval

**Figure 1:**
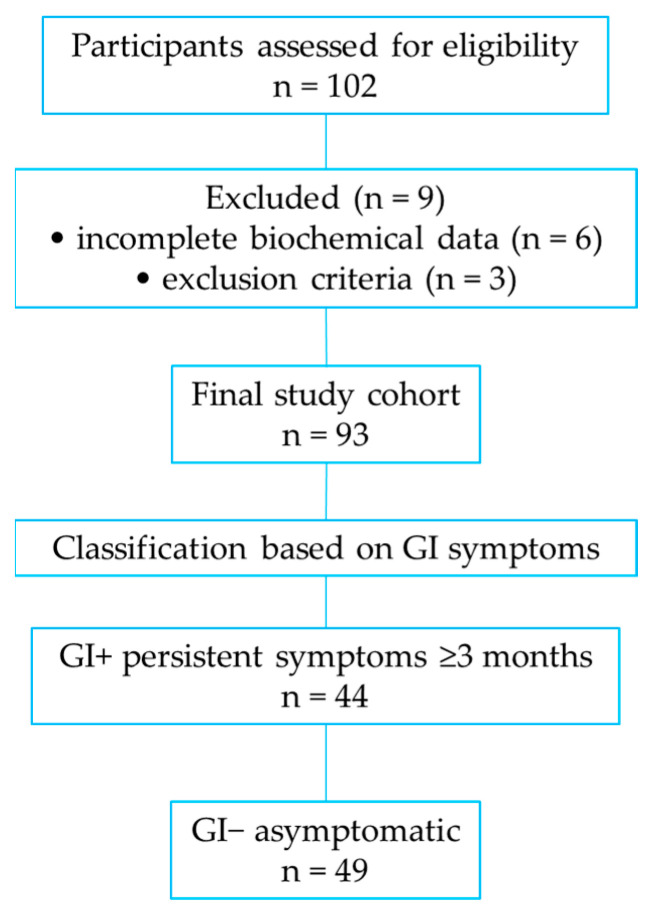
Flowchart of participant inclusion and classification into GI+ and GI− subgroups.

The study was conducted in accordance with the Declaration of Helsinki and the EU General Data Protection Regulation (GDPR 2016/679). Ethical approval was obtained from the Ethics Committee of the University of Oradea (approval code: CEFMF/3; date: 30 October 2023). All participants provided written informed consent prior to inclusion. Data were anonymized and used exclusively for research purposes. The flowchart of participant inclusion and classification into GI+ and GI− subgroups is presented in Figure 1.

## 3. Results

### 3.1. Cohort Demographic Characteristics

The final study cohort comprised 93 adults, with a mean age of 46.8 ± 11.3 years (range: 21–69 years). Females accounted for 59.1% of participants (*n* = 55), while males represented 40.9% (*n* = 38). Missing data were minimal (<5%) and handled by median imputation as described in Methods.

The mean body mass index (BMI) in the overall cohort was 33.43 ± 6.80 kg/m^2^, indicating a predominantly overweight and obese population. According to World Health Organization (WHO) classification, 17.2% of participants were of normal weight (BMI < 25 kg/m^2^), 44.1% were overweight (BMI 25–29.9 kg/m^2^), and 38.7% were classified as obese (BMI ≥ 30 kg/m^2^).

#### Gastrointestinal Symptom Subgroups

Based on the predefined symptom-based classification, 44 participants were categorized as having persistent gastrointestinal symptoms (GI+), while 49 were classified as asymptomatic (GI−). The mean age was comparable between the two groups (48.6 ± 10.9 years in GI+ vs. 45.1 ± 11.6 years in GI−). The median difference in triglyceride levels between GI+ and GI− participants was 106.5 mg/dL.

Females were slightly more represented in the GI+ subgroup (63.6%) compared with the GI− subgroup (55.1%). The mean BMI was 34.15 ± 6.98 kg/m^2^ in GI+ participants and 32.82 ± 6.64 kg/m^2^ in GI− participants. The prevalence of obesity was 45.5% in the GI+ group and 32.7% in the GI− group (Table 1).

### 3.2. Adiposity and Anthropometric Characteristics

Anthropometric evaluation of the cohort (*n* = 93) indicated a predominantly overweight and obese population. The mean body mass index (BMI) was 33.43 ± 6.80 kg/m^2^ (median 33.0; IQR 28.0–38.3). According to World Health Organization categories, 17.2% of participants were of normal weight, 44.1% were overweight, and 38.7% were obese.

Waist circumference was available for 60 participants and showed a mean value of 108.9 ± 14.7 cm (median 108.5; IQR 98.0–118.0), indicating a high prevalence of central adiposity (Table 2).

**Figure 2:**
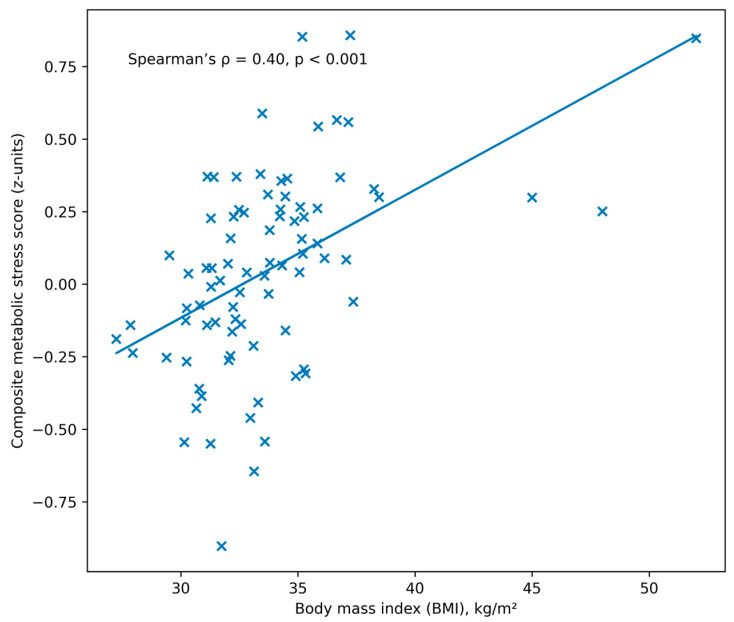
Association between body mass index (BMI) and the composite metabolic stress score in the study cohort (*n* = 93). Spearman correlation analysis demonstrated a moderate positive association (ρ = 0.40, *p* < 0.001). The regression line is shown for visualization purposes only.

Spearman correlation analysis demonstrated a moderate positive association between BMI and the composite metabolic stress score (ρ = 0.40, *p* < 0.001), indicating greater metabolic burden with increasing adiposity (Figure 2).

### 3.3. Glycemic and Lipid Parameters

Glycemic and lipid characteristics of the cohort are presented in Table 3. Fasting plasma glucose showed elevated values (mean 147.7 ± 54.7 mg/dL; median 136 mg/dL, IQR 117–159), while HbA1c averaged 6.35 ± 0.49% (median 6.3%; IQR 6.1–6.8).

The lipid profile reflected an atherogenic pattern, with triglycerides averaging 161.8 ± 105.2 mg/dL (median 127 mg/dL; IQR 88–189) and HDL-cholesterol showing reduced levels (median 41 mg/dL; IQR 36–48). LDL-cholesterol values were more variable and showed no consistent association with metabolic stress.

**Figure 3:**
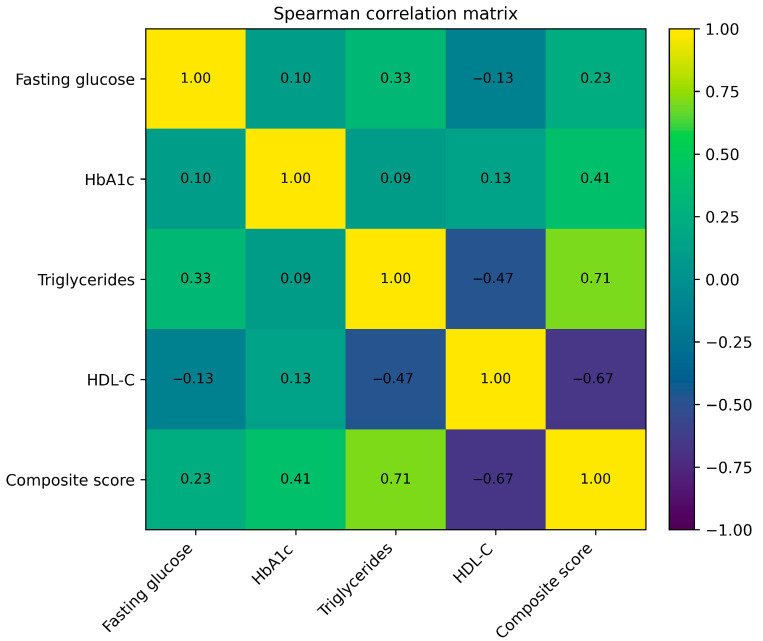
Spearman correlation matrix illustrating associations among glycemic and lipid parameters and the composite metabolic stress score in the study cohort (*n* = 93). Correlation coefficients are shown. The composite score includes triglycerides, HDL-cholesterol, body mass index, and HbA1c.

Spearman correlation analysis demonstrated strong associations between lipid parameters and the composite metabolic stress score. Triglycerides were positively correlated with the score (ρ = 0.62, *p* < 0.001), whereas HDL-cholesterol showed a significant inverse correlation (ρ = −0.51, *p* < 0.001). Fasting glucose showed a weaker but significant positive association (ρ = 0.28, *p* = 0.007), while HbA1c demonstrated a modest correlation (ρ = 0.41, *p* < 0.001). LDL-cholesterol was not significantly correlated with the composite score. Relationships between glycemic, lipid parameters, and the composite metabolic stress score are illustrated in Table 3 and Figure 3.

### 3.4. Cardiovascular Parameters

Descriptive values for cardiovascular parameters are presented in Table 4. Blood pressure measurements were analyzed as independent cardiometabolic variables and were not included in the construction of the composite metabolic stress score.

Systolic blood pressure (SBP) showed a mean value of 136.4 ± 15.2 mmHg, with a median of 135 mmHg (interquartile range [IQR] 126–145). Diastolic blood pressure (DBP) had a mean value of 84.7 ± 9.8 mmHg and a median of 85 mmHg (IQR 78–91). A substantial proportion of participants exhibited blood pressure values above optimal ranges, consistent with elevated cardiometabolic risk in the cohort.

Spearman correlation analysis demonstrated a significant positive association between SBP and the composite metabolic stress score (ρ = 0.54, *p* < 0.001). DBP showed a weaker but statistically significant correlation with the composite score (ρ = 0.26, *p* = 0.03). Heart rate was not significantly associated with the composite metabolic stress score (ρ = 0.10, *p* = 0.40). The distribution of blood pressure values in relation to the composite metabolic stress score is presented in Table 4.

### 3.5. Comparison Between Participants with and Without Gastrointestinal Symptoms

Participants with persistent gastrointestinal symptoms (GI+, *n* = 44) were compared with asymptomatic participants (GI−, *n* = 49) to evaluate differences in cardiometabolic parameters and composite metabolic stress. The GI+ subgroup showed significantly higher values of the composite metabolic stress score compared with the GI− subgroup (median 0.228 vs. −0.323, *p* < 0.001) (Table 5).

Regarding individual metabolic markers, participants in the GI+ group exhibited significantly higher triglyceride levels (median 196.5 vs. 90.0 mg/dL, *p* < 0.001) and lower HDL-cholesterol concentrations (median 36.5 vs. 45.0 mg/dL, *p* < 0.001). Fasting plasma glucose was also significantly higher in the GI+ group (median 141 vs. 127 mg/dL, *p* = 0.008). No statistically significant differences were observed between GI+ and GI− participants for body mass index or HbA1c values (*p* > 0.05 for both).

### 3.6. Integrated Summary of Results

Across the cohort, cardiometabolic parameters showed consistent patterns of association. Adiposity, lipid abnormalities, glycemic markers, and blood pressure were interrelated and aligned with the composite metabolic stress score derived from routine clinical biomarkers.

Triglycerides and HDL-cholesterol displayed the strongest associations with the composite score, while fasting plasma glucose and HbA1c showed more modest correlations. Blood pressure parameters, particularly systolic blood pressure, were also positively associated with the composite metabolic stress score. Subgroup analysis demonstrated that participants with persistent gastrointestinal symptoms exhibited a more adverse cardiometabolic profile, characterized by higher triglyceride levels, lower HDL-cholesterol, higher fasting glucose, and higher composite metabolic stress compared with asymptomatic participants. The correlation matrix summarizing associations among anthropometric, glycemic, lipid, and cardiovascular parameters is shown in Table 6.

## 4. Discussion

The cross-sectional design precludes any inference regarding directionality. Gastrointestinal symptoms may precede, accompany, or result from metabolic dysregulation. The principal finding is that participants reporting chronic gastrointestinal symptoms exhibited a more adverse cardiometabolic profile compared with asymptomatic individuals, characterized primarily by atherogenic dyslipidemia (higher triglycerides and lower HDL-cholesterol) and higher fasting plasma glucose. These alterations were captured by an exploratory composite metabolic stress score derived from routinely available markers, highlighting a pattern of shared metabolic vulnerability rather than a single-organ dysfunction.

### 4.1. Gastrointestinal Symptoms and Cardiometabolic Risk

The association between gastrointestinal symptoms and unfavorable metabolic traits observed in this cohort aligns with accumulating evidence suggesting close links between digestive complaints and metabolic dysregulation. While gastrointestinal symptoms are often considered functional or benign in isolation, our findings indicate that their persistence may coincide with broader cardiometabolic disturbances. Notably, the GI+ subgroup demonstrated significantly higher triglyceride concentrations and lower HDL-cholesterol levels, two key components of atherogenic dyslipidemia, as well as higher fasting glucose levels, despite similar body mass index and HbA1c values compared with GI− participants. Self-reported symptoms do not represent diagnostic evidence of gastrointestinal pathology. Instead, they were treated as a patient-reported phenotype potentially reflecting underlying lifestyle, dietary, or metabolic patterns.

This pattern suggests that gastrointestinal symptom burden may be associated with early or intermediate metabolic alterations that are not fully reflected by long-term glycemic markers or global adiposity alone. Such dissociation underscores the importance of evaluating lipid and short-term glycemic parameters when interpreting metabolic risk in individuals with chronic digestive complaints [14,30].

### 4.2. Composite Metabolic Stress and Multidimensional Risk Patterns

The composite metabolic stress score used in this study was designed to summarize multidimensional cardiometabolic burden using accessible clinical markers rather than to serve as a diagnostic index. Its associations with triglycerides, HDL-cholesterol, fasting glucose, and systolic blood pressure support its internal coherence as a descriptive summary of metabolic stress. Importantly, the higher composite scores observed in GI+ participants reinforce the notion that gastrointestinal symptoms may cluster with broader metabolic risk profiles [30,31]. No a priori power calculation was performed due to the exploratory design. The between-group difference in the composite metabolic stress score appears to be driven predominantly by triglyceride and HDL-cholesterol components, as BMI and HbA1c did not differ significantly between GI+ and GI− participants.

Metabolic disturbances characterized by oxidative stress and low-grade inflammation have been shown to contribute to multisystem tissue vulnerability. Experimental models indicate that metabolic stress can promote systemic inflammatory signaling and organ crosstalk, supporting the plausibility of concurrent gastrointestinal and cardiometabolic alterations.

Such integrative approaches may be particularly useful in exploratory or hypothesis-generating studies, where single biomarkers may fail to capture the complexity of metabolic dysregulation. However, given that the composite score incorporates established cardiometabolic variables, its interpretation should remain descriptive and complementary to individual parameter analysis [32,33,34]. Importantly, the composite metabolic stress score should be considered an exploratory research tool. External validation in larger and independent cohorts is required before any potential clinical application.

### 4.3. Biological Plausibility and the Gut–Metabolism Interface

Although direct measures of gut microbiota composition, intestinal permeability, bile acid metabolism, or hepatic function were not available in this study, the observed associations are biologically plausible within current models of the gut–metabolism interface. Experimental and clinical research has demonstrated that alterations in gut microbial ecology, intestinal barrier function, and diet–microbiota interactions can influence lipid metabolism, glucose homeostasis, and systemic inflammation [35,36]. At a broader epidemiological level, gastrointestinal symptoms have been increasingly recognized as potential indicators of metabolic dysregulation in both European and global populations, particularly in the context of obesity, insulin resistance, and diet-related microbiome alterations.

Within this conceptual framework, persistent gastrointestinal symptoms may reflect underlying functional or microbial disturbances that coexist with cardiometabolic dysregulation. Rather than demonstrating a specific gut–liver axis, our findings are consistent with the hypothesis of shared upstream drivers—such as dietary patterns, low-grade inflammation, or metabolic endotoxemia—that may simultaneously affect gastrointestinal function and metabolic regulation. These mechanisms remain speculative in the context of the present study and warrant direct investigation in future research [37,38].

### 4.4. Clinical Implications

From a clinical perspective, the results suggest that chronic gastrointestinal symptoms should not be viewed solely as isolated functional complaints, particularly in adults with overweight or obesity. The coexistence of digestive symptoms with atherogenic dyslipidemia and impaired fasting glycemia may signal an elevated cardiometabolic risk profile that merits comprehensive metabolic evaluation.

Routine clinical biomarkers, as used in this study, may help identify individuals in whom gastrointestinal symptom burden coincides with metabolic vulnerability, potentially informing early lifestyle or dietary interventions. However, the present findings do not support the use of gastrointestinal symptoms as a diagnostic proxy for metabolic disease, but rather as a contextual factor that may enrich clinical risk assessment [14,30,32,39].

### 4.5. Strengths and Limitations

This study has several strengths. It is based on real-world clinical data and uses standardized metabolic measurements routinely available in clinical practice, enhancing its translational relevance. Gastrointestinal status was defined independently of metabolic variables, thereby avoiding circularity. In addition, the integrative analysis of lipid, glycemic, anthropometric, and cardiovascular parameters provide a multidimensional overview of cardiometabolic risk patterns in a clinical population.

Several limitations should also be acknowledged. First, the cross-sectional design precludes any inference about causality or directionality. Gastrointestinal symptoms may precede, accompany, or result from metabolic dysregulation. Second, gastrointestinal symptoms were self-reported and not supported by standardized diagnostic criteria or objective investigations, which may introduce misclassification bias and reflect perceived symptom burden rather than confirmed gastrointestinal pathology.

Third, potential confounding factors were not systematically controlled. Information on medication use (including lipid-lowering therapy, antihypertensive drugs, and glucose-lowering medications), dietary patterns, smoking status, and physical activity was not consistently available in the clinical records used for this study. These variables may directly influence metabolic parameters and therefore represent potential sources of residual confounding. Consequently, the observed associations should be interpreted cautiously.

Information on medication use (including lipid-lowering, antihypertensive, or antidiabetic treatments), dietary patterns, smoking status, and physical activity was not consistently available. These variables may have influenced metabolic parameters and therefore represent potential residual confounding. Similarly, diabetes status was not formally incorporated as a covariate. In addition, the relatively modest sample size (*n* = 93) limits statistical power, particularly for subgroup analyses. Therefore, the results should be interpreted as exploratory and hypothesis-generating rather than definitive evidence of cardiometabolic associations with gastrointestinal symptoms.

Fourth, the absence of direct gastrointestinal or hepatic biomarkers (e.g., microbiome profiling, intestinal permeability markers, or bile acid analyses) limits mechanistic interpretation. The composite metabolic stress score, while transparently constructed, is exploratory and not a validated diagnostic tool.

Finally, the single-center convenience sample and the predominance of overweight and obese participants limit generalizability to the broader population. However, this also allowed focused exploration within a metabolically vulnerable group frequently encountered in clinical nutrition practice. The modest sample size further supports interpreting the findings as hypothesis-generating rather than definitive.

### 4.6. Future Directions

Future studies should incorporate longitudinal designs and objective gastrointestinal phenotyping, including microbiome profiling, intestinal permeability markers, bile acid analysis, and hepatic imaging or biomarkers. Such approaches would allow direct testing of the hypothesized links between gastrointestinal function and cardiometabolic regulation and clarify whether gastrointestinal symptoms precede, accompany, or result from metabolic dysregulation.

In conclusion, persistent gastrointestinal symptoms in adults were associated with a more adverse cardiometabolic profile characterized by atherogenic dyslipidemia and higher fasting glucose levels. An exploratory composite metabolic stress score derived from routine biomarkers summarized this pattern effectively. These findings support the biological plausibility of shared metabolic vulnerability between gastrointestinal symptom burden and cardiometabolic risk, while underscoring the need for mechanistic and longitudinal studies to further elucidate these relationships.

## 5. Conclusions

In this adult cohort, persistent GI symptoms were associated with a more adverse cardiometabolic pattern, primarily driven by atherogenic dyslipidemia (higher TG and lower HDL-C) and higher fasting glucose. An exploratory composite metabolic stress score based on routine biomarkers summarized this burden and differed significantly between symptom-defined subgroups. This exploratory study does not propose gastrointestinal symptoms as diagnostic markers but as clinical context signals warranting further investigation.

Given the cross-sectional design and symptom-based GI classification, these findings should be interpreted as hypothesis-generating and require validation using objective gastrointestinal phenotyping and mechanistic biomarkers in prospective studies.

## Figures and Tables

**Table 1:** Demographic and Anthropometric Characteristics of the Study Cohort According to Gastrointestinal Symptom Status.

Parameter	Total (*n* = 93)	GI+ (*n* = 44)	GI− (*n* = 49)	*p*-Value
Age (years), mean ± SD	46.8 ± 11.3	48.6 ± 10.9	45.1 ± 11.6	0.115
Female sex, *n* (%)	55 (59.1)	28 (63.6)	27 (55.1)	0.068
BMI (kg/m^2^), mean ± SD	33.43 ± 6.80	34.15 ± 6.98	32.82 ± 6.64	0.503
Normal weight, *n* (%)	16 (17.2)	5 (11.4)	11 (22.4)	—
Overweight, *n* (%)	41 (44.1)	19 (43.2)	22 (44.9)	—
Obesity, *n* (%)	36 (38.7)	20 (45.5)	16 (32.7)	—

BMI, body mass index; SD, standard deviation; GI+, participants with persistent gastrointestinal symptoms; GI−, asymptomatic participants. Weight categories were defined according to World Health Organization criteria: normal weight (BMI < 25 kg/m^2^), overweight (25–29.9 kg/m^2^), and obesity (≥30 kg/m^2^). Continuous variables were compared using the Mann–Whitney U test. Categorical variables were compared using the chi-square test where applicable. Weight categories are reported descriptively because expected cell counts were insufficient for robust chi-square testing.

**Table 2:** Anthropometric Characteristics of the Total Study Cohort (*n* = 93).

Parameter	Mean ± SD	Median (IQR)	Prevalence (%)
BMI (kg/m^2^)	33.43 ± 6.80	33.0 (28.0–38.3)	—
Waist circumference (cm) *	108.9 ± 14.7	108.5 (98.0–118.0)	—
Obesity (BMI ≥ 30 kg/m^2^)	—	—	38.7

BMI, body mass index; SD, standard deviation; IQR, interquartile range. * Waist circumference was available for 60 participants.

**Table 3:** Glycemic and Lipid Characteristics of the Study Cohort (*n* = 93).

Parameter	Mean ± SD	Median (IQR)	Reference Range *
Fasting plasma glucose (mg/dL)	147.7 ± 54.7	136 (117–159)	70–99
HbA1c (%)	6.35 ± 0.49	6.3 (6.1–6.8)	<5.7
Triglycerides (mg/dL)	161.8 ± 105.2	127 (88–189)	<150
HDL-cholesterol (mg/dL)	42.4 ± 9.5	41 (36–48)	>50 (F)/>40 (M)

SD, standard deviation; IQR, interquartile range; HbA1c, glycated hemoglobin; HDL-C, high-density lipoprotein cholesterol. * Reference ranges are indicative and based on American Diabetes Association and ESC/EAS guideline recommendations.

**Table 4:** Cardiovascular Characteristics of the Study Cohort (*n* = 93).

Parameter	Mean ± SD	Median (IQR)	Reference Range *
Systolic blood pressure (mmHg)	136.4 ± 15.2	135 (126–145)	<130
Diastolic blood pressure (mmHg)	84.7 ± 9.8	85 (78–91)	<85
Heart rate (beats/min)	76.3 ± 9.1	75 (70–82)	60–80
Composite metabolic stress score (z-units)	0.00 ± 1.00	0.02 (–0.61–0.75)	—

SD, standard deviation; IQR, interquartile range. * Reference ranges are based on ESC/ESH guideline recommendations for blood pressure and standard clinical references for heart rate.

**Table 5:** Comparison of Cardiometabolic Parameters Between Participants With (GI+) and Without (GI−) Gastrointestinal Symptoms.

Parameter	GI+ (*n* = 44) Median	GI− (*n* = 49) Median	*p*-Value
Composite metabolic stress score (z-units)	0.228	−0.323	<0.001
Triglycerides (mg/dL)	196.5	90.0	<0.001
HDL-cholesterol (mg/dL)	36.5	45.0	<0.001
Fasting plasma glucose (mg/dL)	141	127	0.008
HbA1c (%)	6.35	6.30	0.693
BMI (kg/m^2^)	33.80	33.00	0.503

HDL-C, high-density lipoprotein cholesterol; BMI, body mass index; GI+, participants with persistent gastrointestinal symptoms; GI−, asymptomatic participants. Group comparisons were performed using the Mann–Whitney U test. Median values are reported for non-normally distributed variables.

**Table 6:** Spearman Correlations Between Cardiometabolic Parameters and the Composite Metabolic Stress Score (|ρ| ≥ 0.30).

Variable 1	Variable 2	Spearman’s ρ
Composite metabolic stress score (z-units)	Triglycerides (mg/dL)	0.71
Composite metabolic stress score (z-units)	HDL-cholesterol (mg/dL)	−0.67
Composite metabolic stress score (z-units)	Body mass index (kg/m^2^)	0.40
Composite metabolic stress score (z-units)	HbA1c (%)	0.41
Systolic blood pressure (mmHg)	Composite metabolic stress score (z-units)	0.54

BMI, body mass index; HDL-C, high-density lipoprotein cholesterol. Spearman correlation coefficients (ρ) were calculated for the entire cohort (*n* = 93). Only correlations with |ρ| ≥ 0.30 and *p* < 0.05 are shown.

## Data Availability

The data presented in this study are openly available in Figshare at https://doi.org/10.6084/m9.figshare.6025748.v1. Additional raw data can be made available from the corresponding author upon reasonable request, in compliance with national data protection regulations.

## Abbreviations

The following abbreviations are used in this manuscript:

Abbreviation	Full Term
ADA	American Diabetes Association
BMI	Body mass index
CC BY	Creative Commons Attribution
DBP	Diastolic blood pressure
ESC	European Society of Cardiology
ESC/EAS	European Society of Cardiology/European Atherosclerosis Society
ESC/ESH	European Society of Cardiology/European Society of Hypertension
EU	European Union
GDPR	General Data Protection Regulation
GI	Gastrointestinal
GI+	Participants with persistent gastrointestinal symptoms
GI−	Asymptomatic participants
HbA1c	Glycated hemoglobin
HDL-C	High-density lipoprotein cholesterol
IQR	Interquartile range
LDL-C	Low-density lipoprotein cholesterol
SBP	Systolic blood pressure
SD	Standard deviation
SPSS	Statistical Package for the Social Sciences
TG	Triglycerides
WC	Waist circumference
WHO	World Health Organization

## References

[1] Zulet Fraile P., Lizancos Castro A., Andía Melero V., González Antigüedad C., Monereo Megías S., Calvo Revilla S. (2019). [Relationship of body composition measured by dexa with lifestyle and satisfaction with body image in university students]. Nutr. Hosp..

[2] Miller A., McNamara J., Hummel S.L., Konerman M.C., Tincopa M.A. (2020). Prevalence and staging of non-alcoholic fatty liver disease among patients with heart failure with preserved ejection fraction. Sci. Rep..

[3] Bryant R.V., Schultz C.G., Ooi S., Goess C., Costello S.P., Vincent A.D., Schoeman S.N., Lim A., Bartholomeusz F.D., Travis S.P.L. (2018). Obesity in inflammatory bowel disease: Gains in adiposity despite high prevalence of myopenia and osteopenia. Nutrients.

[4] ElSayed N.A., McCoy R.G., Aleppo G., Balapattabi K., Beverly E.A., Early K.B., Bruemmer D., Echouffo-Tcheugui J.B., Ekhlaspour L., American Diabetes Association Professional Practice Committee (2024). 16. Diabetes care in the hospital: Standards of care in diabetes—2025. Diabetes Care.

[5] Al-Hassani I., Khan N.A., Elmenyar E., Al-Hassani A., Rizoli S., Al-Thani H., El-Menyar A. (2024). The interaction and implication of stress-induced hyperglycemia and cytokine release following traumatic injury: A structured scoping review. Diagnostics.

[6] Beckman J.A., Creager M.A., Libby P. (2002). Diabetes and atherosclerosis: Epidemiology, pathophysiology, and management. JAMA.

[7] Mach F., Koskinas K.C., Roeters van Lennep J.E., Tokgözoğlu L., Badimon L., Baigent C., Benn M., Binder C.J., Catapano A.L., De Backer G.G. (2025). 2025 focused update of the 2019 esc/eas guidelines for the management of dyslipidaemias: Developed by the task force for the management of dyslipidaemias of the european society of cardiology (esc) and the european atherosclerosis society (eas). Eur. Heart J..

[8] Lu S., Xie Q., Kuang M., Hu C., Li X., Yang H., Sheng G., Xie G., Zou Y. (2023). Lipid metabolism, bmi and the risk of nonalcoholic fatty liver disease in the general population: Evidence from a mediation analysis. J. Transl. Med..

[9] Omolekulo T.E., Michael O.S., Olatunji L.A. (2019). Dipeptidyl peptidase-4 inhibition protects the liver of insulin-resistant female rats against triglyceride accumulation by suppressing uric acid. Biomed. Pharmacother..

[10] McCarthy C.P., Bruno R.M., McEvoy J.W., Touyz R.M. (2025). 2024 Esc Guidelines for the Management of Elevated Blood Pressure and Hypertension: What is New in Pharmacotherapy?.

[11] Abbud Z.A., Shindler D.M., Wilson A.C., Kostis J.B. (1995). Effect of diabetes mellitus on short- and long-term mortality rates of patients with acute myocardial infarction: A statewide study. Myocardial infarction data acquisition system study group. Am. Heart J..

[12] Hernández-Mariano J., Baltazar-Reyes M.C., Salazar-Martínez E., Cupul-Uicab L.A. (2022). Exposure to the pesticide ddt and risk of diabetes and hypertension: Systematic review and meta-analysis of prospective studies. Int. J. Hyg. Environ. Health.

[13] Phaniendra A., Jestadi D.B., Periyasamy L. (2015). Free radicals: Properties, sources, targets, and their implication in various diseases. Indian J. Clin. Biochem..

[14] Hsu C.L., Schnabl B. (2023). The gut-liver axis and gut microbiota in health and liver disease. Nat. Rev. Microbiol..

[15] Liu Y.J., Kimura M., Li X., Sulc J., Wang Q., Rodríguez-López S., Scantlebery A.M., Strotjohann K., Gallart-Ayala H., Vijayakumar A. (2025). Acmsd inhibition corrects fibrosis, inflammation, and DNA damage in masld/mash. J. Hepatol..

[16] Wang D., Miao J., Zhang L., Zhang L. (2025). Research advances in the diagnosis and treatment of masld/mash. Ann. Med..

[17] Wang X., Zhang L., Dong B. (2024). Molecular mechanisms in masld/mash related hcc. Hepatology.

[18] Wang X., Klaassen C.D., Chen X., Zhang Y. (2025). Pathological and therapeutic roles of bile acid metabolism and signaling in hepatocellular carcinoma: Insights from human and mouse studies. Pharmacol. Rev..

[19] Perez M.J., Briz O. (2009). Bile-acid-induced cell injury and protection. World J. Gastroenterol..

[20] Ayivi-Tosuh S.M., Dofuor A.K., Yamoah J.A.A., Gayi B.K., Aiduenu A.F., Akomea A., Anovunga S.A., Ekloh W., Basing L.A. (2026). Gut-microbiome interactions: Characterization, therapeutic implications and machine learning. Sage Open Pathol..

[21] Wachsmuth H.R., Weninger S.N., Duca F.A. (2022). Role of the gut–brain axis in energy and glucose metabolism. Exp. Mol. Med..

[22] De Filippis A., Ullah H., Baldi A., Dacrema M., Esposito C., Garzarella E.U., Santarcangelo C., Tantipongpiradet A., Daglia M. (2020). Gastrointestinal disorders and metabolic syndrome: Dysbiosis as a key link and common bioactive dietary components useful for their treatment. Int. J. Mol. Sci..

[23] Jones H., Alpini G., Francis H. (2015). Bile acid signaling and biliary functions. Acta Pharm. Sin. B.

[24] Ahmad M.F., Ahmad F.A., Alsayegh A.A., Zeyaullah M., AlShahrani A.M., Muzammil K., Saati A.A., Wahab S., Elbendary E.Y., Kambal N. (2024). Pesticides impacts on human health and the environment with their mechanisms of action and possible countermeasures. Heliyon.

[25] Ezenabor E.H., Adeyemi A.A., Adeyemi O.S. (2024). Gut microbiota and metabolic syndrome: Relationships and opportunities for new therapeutic strategies. Scientifica.

[26] Amiri P., Hosseini S.A., Ghaffari S., Tutunchi H., Ghaffari S., Mosharkesh E., Asghari S., Roshanravan N. (2022). Role of butyrate, a gut microbiota derived metabolite, in cardiovascular diseases: A comprehensive narrative review. Front. Pharmacol..

[27] Paduraru L., Vesa C.M., Popoviciu M.S., Ghitea T.C., Zaha D.C. (2025). Interrelationship between dyslipidemia and hyperuricemia in patients with uncontrolled type 2 diabetes: Clinical implications and a risk identification algorithm. Healthcare.

[28] Moldovan A.F., Moga I., Moga T., Ghitea E.C., Babes K., Ghitea T.C. (2023). Assessing the risk of stroke in the elderly in the context of long-covid, followed through the lens of family medicine. In Vivo.

[29] Dhondge R.H., Agrawal S., Patil R., Kadu A., Kothari M. (2024). A comprehensive review of metabolic syndrome and its role in cardiovascular disease and type 2 diabetes mellitus: Mechanisms, risk factors, and management. Cureus.

[30] Fousekis F.S., Mpakogiannis K., Lianos G.D., Antonelli E., Bassotti G., Katsanos K.H. (2025). Gut–liver axis, microbiota, bile acids, and immune response in pathogenesis of primary sclerosing cholangitis: An overview. J. Clin. Med..

[31] Zhu H.-H., Zuo S.T., Wang J. (2025). Research advances in the association between gut microbiota and hepatic alveolar echinococcosis: A review study. Hepat. Mon..

[32] Ali A., AlHussaini K.I. (2024). Pesticides: Unintended impact on the hidden world of gut microbiota. Metabolites.

[33] Matsuzaki R., Gunnigle E., Geissen V., Clarke G., Nagpal J., Cryan J.F. (2023). Pesticide exposure and the microbiota-gut-brain axis. ISME J..

[34] Stroia C.M., Ghitea T.C., Vrânceanu M., Mureșan M., Bimbo-Szuhai E., Pallag C.R., Pallag A. (2024). Relationship between vitamin d3 deficiency, metabolic syndrome and vdr, gc, and cyp2r1 gene polymorphisms. Nutrients.

[35] Hoca E., Cangir B., Ahbab S., Şahin S.İ., Çiftçi Öztürk E., Urvasızoğlu A.Ö., Kalaycı N., Engin İ., Ataoğlu H.E. (2025). The triglyceride/hdl ratio as a non-invasive marker for early-stage nafld: A retrospective cross-sectional study of 2588 patients. Diagnostics.

[36] Kyhl L.K., Nordestgaard B.G., Tybjærg-Hansen A., Nielsen S.F. (2025). Metabolic dysfunction-associated steatotic liver disease, cardiometabolic risk factors, and cardiovascular disease. Atherosclerosis.

[37] Ciardullo S., Grassi G., Mancia G., Perseghin G. (2022). Nonalcoholic fatty liver disease and risk of incident hypertension: A systematic review and meta-analysis. Eur. J. Gastroenterol. Hepatol..

[38] Sonaglioni A., Cerini F., Fagiani V., Nicolosi G.L., Rumi M.G., Lombardo M., Muti P. (2025). Effect of metabolic dysfunction-associated steatotic liver disease (masld) on left ventricular mechanics in patients without overt cardiac disease: A systematic review and meta-analysis. J. Clin. Med..

[39] Tomuța R.A., Moldovan A.F., Matiș L., Maris L., Ghitea T.C., Banica F. (2025). The paradox of clean eating: Neuroactive dysbiosis and pesticide residues in fruit- and vegetable-based diets. Toxics.

